# Interlayer Interactions and Macroscopic Property Calculations of Squaric-Acid-Linked Zwitterionic Covalent Organic Frameworks: Structures, Photocatalytic Carrier Transport, and a DFT Study

**DOI:** 10.3390/molecules29122739

**Published:** 2024-06-08

**Authors:** Gaojie Yan, Xiaojie Zhang

**Affiliations:** Hebei Key Laboratory of Functional Polymers, Department of Polymer Materials and Engineering, Hebei University of Technology, Tianjin 300401, China; 202021501025@stu.hebut.edu.cn

**Keywords:** zwitterionic covalent organic frameworks, density functional theory, weak interactions, macroscopic carrier transport, photocatalytic materials

## Abstract

Squaric-acid-linked zwitterionic covalent organic frameworks (Z-COFs), assembled through interlayer interactions, are emerging as potential materials in the field of photocatalysis. However, the study of their interlayer interactions has been largely overlooked. To address this, this work systematically calculated interlayer interactions via density functional theory (DFT) and analyzed the differences in interlayer interactions of different structures of Z-COFs through interlayer slippage, planarity, and an independent gradient model based on the Hirshfeld partition (IGMH). Furthermore, it revealed the relationship between the interactions and the macroscopic photocatalytic carrier transport performance of the material. The results indicated that both preventing interlayer slippage and enhancing planarity can enhance the interlayer interactions of Z-COFs, thereby improving their macroscopic carrier transport performance in photocatalysis.

## 1. Introduction

The burgeoning field of materials science is continuously unveiling novel structures with unprecedented properties, and among these, covalent organic frameworks (COFs) stand out for their potential in a myriad of applications [1,2,3,4,5]. Extending the versatility of COFs, zwitterionic covalent organic frameworks (Z-COFs) represent a fascinating subclass that incorporates both positively and negatively charged functional groups within a single, well-defined structure. These unique materials exhibit a delicate balance of charges, endowing them with exceptional properties that are of keen interest for various technological applications. In addition, in the field of photocatalysis, Z-COFs exhibit significant advantages [6,7,8]. Besides regulating the basic units of Z-COFs, the photocatalytic process can be controlled by introducing specific chemical groups, which are the effects of light absorption and charge separation [9,10]. Due to the stable nanochannels of Z-COF materials, precise regulation of their pore structure can achieve specific pore sizes and specific surface areas. This allows for rapid diffusion of charge carriers, which is conducive to improving the migration rate of charge carriers.

However, the layers of Z-COF materials are connected through non-covalent interactions. The accurate stacking order of these layers is crucial for the photocatalytic performance of these polymer materials. The magnitude of interlayer interactions directly affects the stability of COFs. Weak interlayer interactions can lead to various stacking geometries within Z-COFs, reducing the crystallinity of Z-COF materials, which is detrimental to charge transport and charge carrier recombination, thus affecting photocatalytic efficiency. Concurrently, density function theory (DFT) calculations are becoming a popular and powerful tool to study materials science [11,12,13,14,15]. It can offer unparalleled insights into the electronic structure and properties of materials at the atomic level. DFT enables researchers to predict and manipulate the behavior of materials under various conditions, which is crucial for the design and development of new materials with tailored properties.

**Scheme 1:**
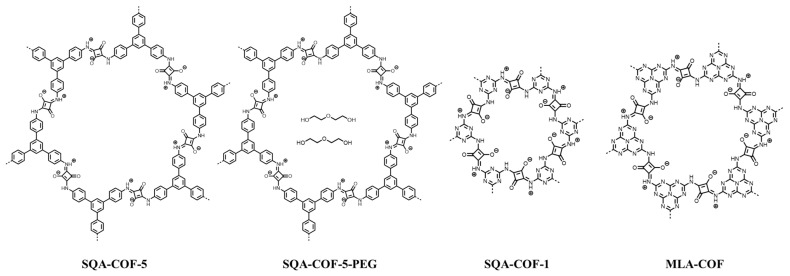
Structures of SQA-COF-5, SQA-COF-5-PEG, SAQ-COF-1, and MLA-COF.

Hence, we selected two reported Z-COFs, namely SQA-COF-1 [16] and SQA-COF-5 [17] (Scheme 1). Additionally, a low molecular weight polyethylene glycol (PEG, hereinafter referred to as SQA-COF-5-PEG) was inserted into SQA-COF-5 to study its effect on interlayer interactions. In the reported work [18], PEG can make coaxial stacking of two-dimensional COFs stable. Furthermore, a Z-COF with a heptazine ring as the nodal unit (hereinafter referred to as MLA-COF) was designed to investigate the influence of structure on interlayer interaction and macroscopic performance. Interlayer interaction calculations were performed for the aforementioned four types of Z-COFs, with a detailed study of the effects of interlayer interactions on slippage, planarity, and weak interactions. The macroscopic properties related to photocatalysis (such as photocatalytic carrier transport, bandgap, and the polarized electric field strength) for the four Z-COFs were also calculated. We believe that calculating the interlayer interactions of Z-COFs can help to determine the stability and crystallinity of Z-COF materials. It furthers our understanding of the contribution of interlayer interactions to photocatalytic properties, establishes the relationship between aggregated state structure and macroscopic performance, and refines the design concept of COF materials for high-performance photocatalysts based on localized space polarization-induced efficient charge separation.

## 2. Results and Discussions

### 2.1. Slippage

The crystal structure models of SQA-COF-5, SQA-COF-5-PEG, SQA-COF-1, and MLA-COF were established and optimized, with their unit cell parameters shown in Table 1 and Figure S1. For the cell parameters, it can be seen that the introduction of PEG does not significantly enhance the π-π stacking effect of SQA-COF-5. However, the introduction of planar and conjugated units rich in nitrogen atoms, such as SQA-COF-1 and MLA-COF, can significantly enhance the symmetry of the space group. With the addition of SQA-COF-1 and MLA-COF, the space group without symmetry P1, is elevated to the higher symmetry P6/m space group, which is more conducive to interlayer stacking. The most significant change is the reduction in interlayer spacing, which better utilizes the anchoring effect of π-π stacking to stabilize the crystal domains. It is worth noting that the distance of the π-π interaction has a certain range, generally between 3.5 Å and 4.0 Å. Therefore, the interlayer spacing of MLA-COF (3.427 Å) compared to SQA-COF-1 (3.428 Å) shows a smaller change and a minimal increase. However, MLA-COF with a stronger stacking effect has better stability in π-π interaction, which is also evident in the subsequent slippage studies. It is anticipated that its macroscopic performance will also be superior to SQA-COF-1 to a certain extent.

**Figure 1:**
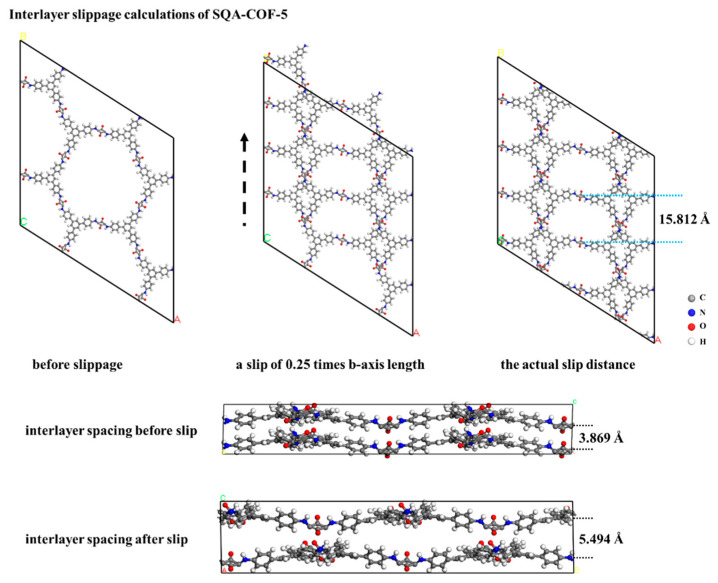
SQA-COF-5 layer distance before and after slippage and actual slip distance.

**Figure 2:**
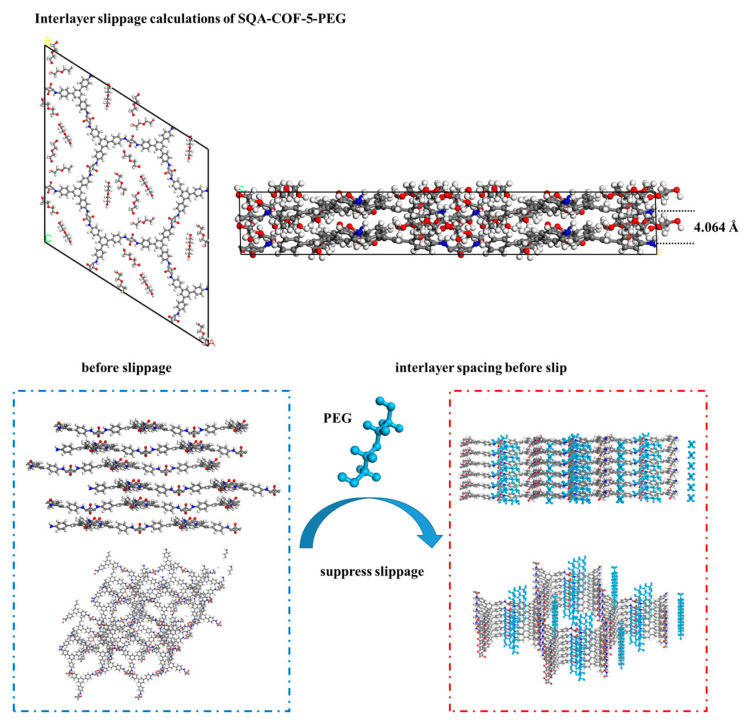
SQA-COF-5-PEG layer distance before slippage and effect of PEG suppressing slippage.

**Figure 3:**
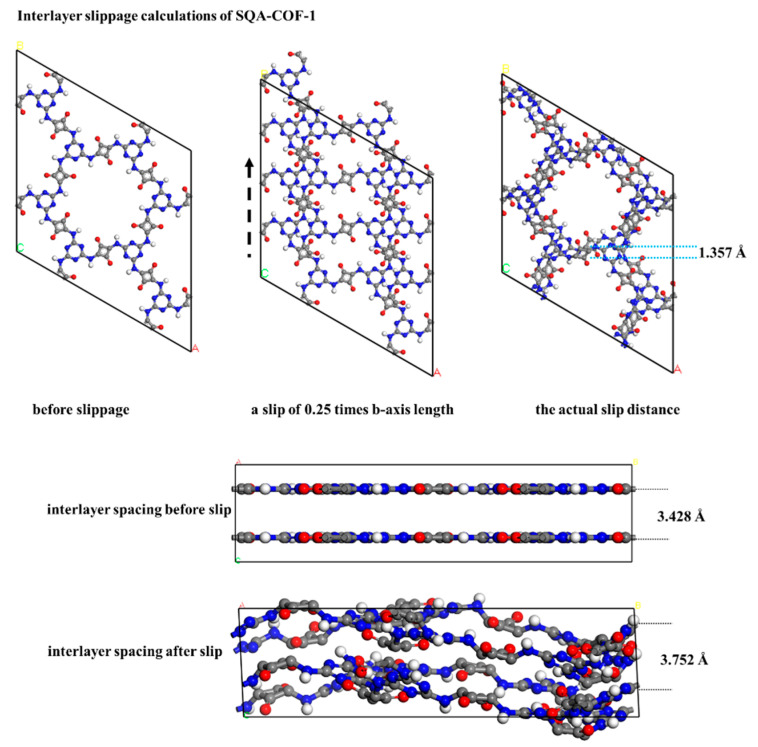
SQA-COF-1 layer distance before and after slippage and actual slip distance.

**Figure 4:**
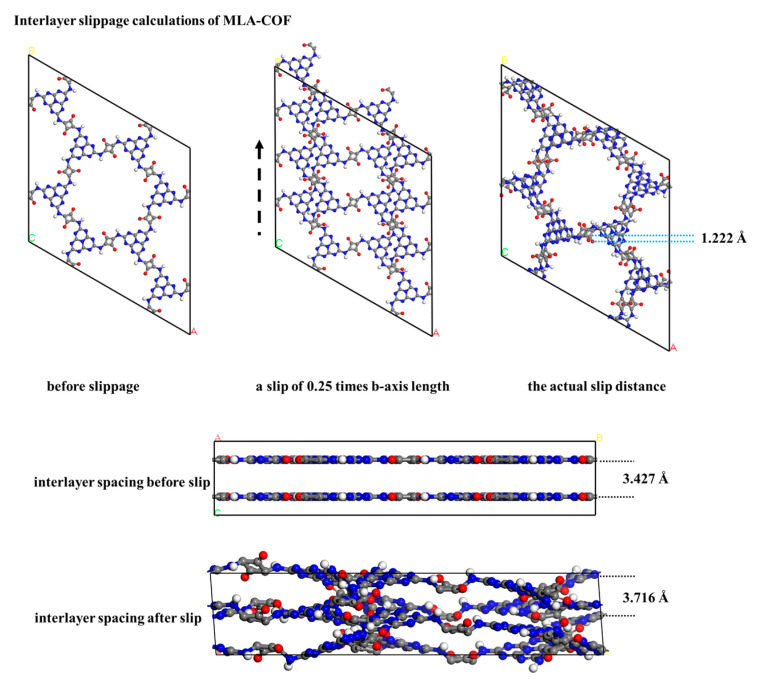
MLA-COF layer distance before and after slippage and actual slip distance.

To demonstrate the anchoring effect of π-π stacking, slippage studies were conducted on the (001) crystal plane of SQA-COF-5, SQA-COF-5-PEG, SQA-COF-1, and MLA-COF. Initially, a 2 × 2 × 2 supercell was established based on the primitive cell. On this supercell, a layer was translated along the *b*-axis by a distance of 0.25 times the *b*-axis length, and the parameters after slippage were then calculated. The layer spacing before and after slippage and the actual slip distance L2 for SQA-COF-5, SQA-COF-5-PEG, SQA-COF-1, and MLA-COF are shown in Figure 1, Figure 2, Figure 3 and Figure 4.

**Figure 5:**
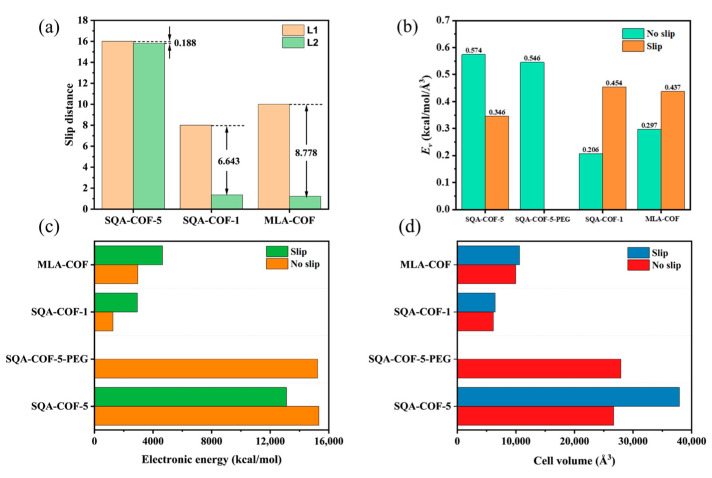
(**a**) Expected and actual slippage distance of SQA-COF-5, SQA-COF-1 and MLA-COF; (**b**) specific volume electron energy, (**c**) electron energy and (**d**) unit cell volume of SQA-COF-5, SQA-COF-5-PEG, SQA-COF-1 and MLA-COF.

The supercell parameters before slippage for SQA-COF-5, SQA-COF-5-PEG, SQA-COF-1, and MLA-COF are shown in Table 2, and the parameters after slippage are shown in Table 3. A summary plot of the parameters before and after slippage is presented in Figure S2. It can be observed that the changes in the *a*-axis, *b*-axis, and the three crystal angles are minimal for SQA-COF-5, SQA-COF-1, and MLA-COF before and after slippage. The most significant change is in the *c*-axis interlayer spacing. The layer spacing (*c*-axis) change for SQA-COF-1 and MLA-COF, which have better π-π stacking interactions, is much smaller than that for SQA-COF-5 (as shown in Figure 5a). At the same time, the expected slip distance (L1) and the actual slip distance (L2) also differ greatly. The larger the difference between L1 and L2, the better the slippage suppression effect. In the previous analysis, it was pointed out that MLA-COF with a stronger stacking effect has better stability in π-π interactions than SQA-COF-1, which is also confirmed in this slippage study. It is worth noting that MLA-COF has a smaller actual slip distance (1.222 Å) than SQA-COF-1 (1.357 Å), and the difference between L1 and L2 is also larger for MLA-COF. Meanwhile, the interlayer spacing after slippage for MLA-COF (7.432 Å) is also smaller than that for SQA-COF-1 (7.504 Å).

Finally, the concept of specific volume electron energy is defined to support the previous arguments and to provide a unified explanation. From the above analysis, it can be concluded that during the slippage process, both the volume of the unit cell and the energy undergo significant changes. Therefore, it is necessary to consider both factors. The specific volume electron energy is defined to evaluate the difficulty of slippage. Figure 5b–d show the relative sizes of the electron energy, unit cell volume, and specific volume electron energy of the following four Z-COFs: SQA-COF-5, SQA-COF-5-PEG, SQA-COF-1, and MLA-COF. The specific data are summarized in Table S1. The results show that the specific volume electron energy (*E*_V_) of SQA-COF-5 decreases after slippage, indicating that slippage makes its crystal domains more stable. In contrast, the *E*_V_ of SQA-COF-1 and MLA-COF increases after slippage, making it difficult for them to slip. This indicates that their π-π interaction anchoring effect can effectively limit slippage. It is worth noting that the concept of *E*_V_ is suitable for comparing before and after slippage of the same material because it is related to the number of atoms and the size of the unit cell. Therefore, it is not very suitable for comparing SQA-COF-5, MLA-COF, and SQA-COF-1 with each other. However, it can be approximately compared when the unit cell volume and the number of atoms is equal. For example, the *E*_V_ of SQA-COF-5-PEG is lower than that of SQA-COF-5, which also indicates to some extent that the addition of PEG is beneficial for stabilizing the crystal domains.

### 2.2. Planarity and IGMH Analysis

**Figure 6:**
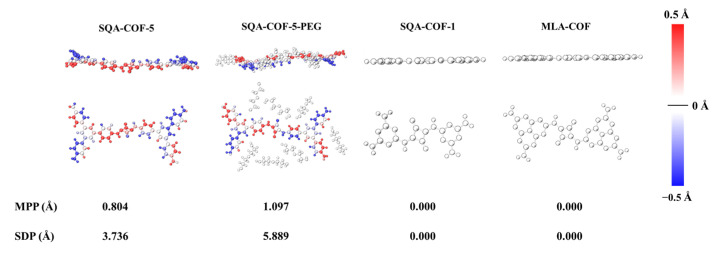
Color map of signed distance values for SQA-COF-5, SQA-COF-5-PEG, SQA-COF-1, and MLA-COF (**left** to **right**) and associated MPP and SDP values.

Planarity is a very important parameter for structures with π-π stacking interactions [19]. The molecular planarity parameter (MPP) and span of deviation from plane (SDP) were specific to judge the planarity. For both MPP and SDP, the smaller the better, with MPP reflecting the overall stability of the region and SDP reflecting the degree of atomic deviation on both sides of the fitted plane. In Figure 6, it can be seen that the MPP and SDP values of SQA-COF-5 and SQA-COF-5-PEG are larger than those of SQA-COF-1 and MLA-COF, indicating poorer planarity for SQA-COF-5 and SQA-COF-5-PEG. Meanwhile, after the introduction of PEG, both MPP and SDP increase, indicating that the added PEG does not favor the improvement of planarity. It is worth noting that after the introduction of the planar and conjugated units triazine and melamine, the MPP and SDP of SQA-COF-1 and MLA-COF are both zero, representing an ideal absolute plane. This indicates that the introduction of conjugated amine building units is beneficial for enhancing the planarity of the benzene series COFs.

**Figure 7:**
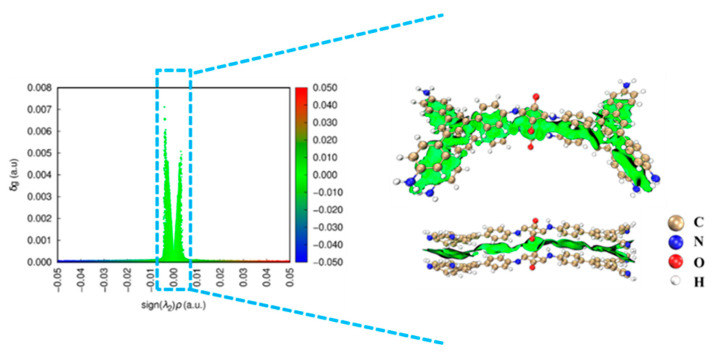
Profiles of electron density gradient difference vs. electron density of SQA-COF-5 (**left**) and its associated isosurface map (**right**).

**Figure 8:**
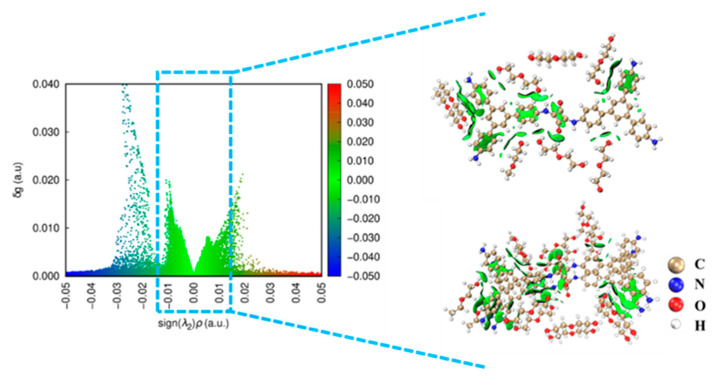
Profiles of electron density gradient difference vs. electron density of SQA-COF-5-PEG (**left**) and its associated isosurface map (**right**).

**Figure 9:**
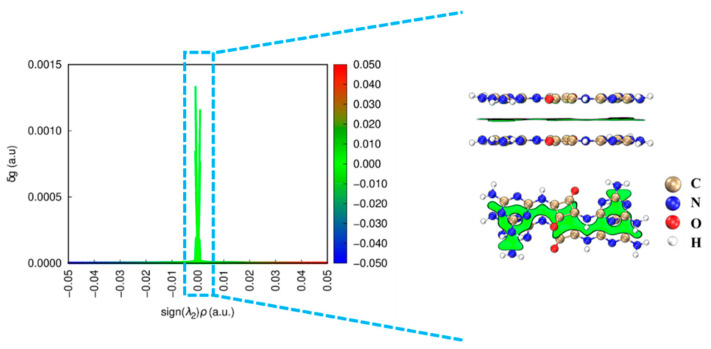
Profiles of electron density gradient difference vs. electron density of SQA-COF-1 (**left**) and its associated isosurface map (**right**).

**Figure 10:**
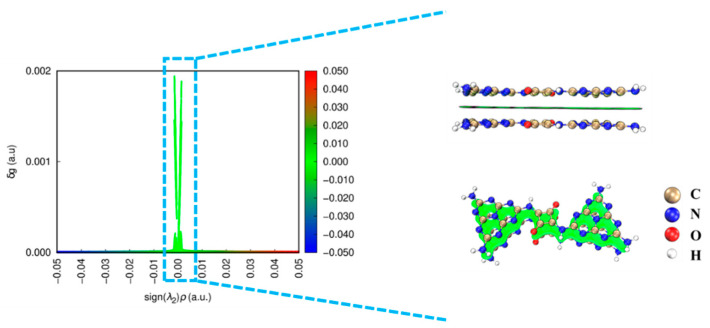
Profiles of electron density gradient difference vs. electron density of MLA-COF (**left**) and its associated isosurface map (**right**).

Before calculating the interactions, the primitive cell was expanded to a 1 × 1 × 2 supercell, and the wave function was obtained using the xtb 6.4.1 software at the GFN2 computational level. Then, Multiwfn 3.8 was used to analyze the weak interactions within the system based on the independent gradient model based on Hirshfeld partition (IGMH). It was a more universal new method proposed by Lu Tian et al. [20]. In 2008, Grimme revealed that the essence of the π-π interaction is the dispersion interaction, a type of van der Waals force, corresponding to the part of the IGMH theory where the electron density is approximately zero [21]. Figure S3 shows the basis for judgment in the IGMH method. IGMH analysis was performed on SQA-COF-5, SQA-COF-5-PEG, SQA-COF-1, and MLA-COF (Figure 7, Figure 8, Figure 9 and Figure 10). In Figure 7, the relationship between the electron density gradient difference (*δ*_g_) and the electron density shows that *δ*_g_ is very small at *ρ* = 0, indicating that SQA-COF-5 has a weaker π-π interaction. Its isosurface map also shows that the π-π interaction in SQA-COF-5 is non-planar, further confirming the weak π-π interaction of SQA-COF-5. Although the electron density gradient difference *δ*_g_ at *ρ* = 0 for SQA-COF-5 is small, it is larger than that for SQA-COF-5-PEG, suggesting that SQA-COF-5 has a better stacking effect than SQA-COF-5-PEG. Notably, in Figure 8, the relationship between the electron density gradient difference and the electron density shows a significant *δ*_g_ value on both sides of *ρ* = 0 (−0.01~0.01 a.u.), and combined with its isosurface map, it is evident that hydrogen bonds are formed in SQA-COF-5-PEG, which also contributes to the stabilization of the COF framework from another aspect. As shown in Figure 8 and Figure 10, both SQA-COF-1 and MLA-COF have concentrated *δ*_g_ values at *ρ* = 0, and their isosurface maps indicate that they have very strong π-π interactions between layers, which can stabilize the COF framework.

**Figure 11:**
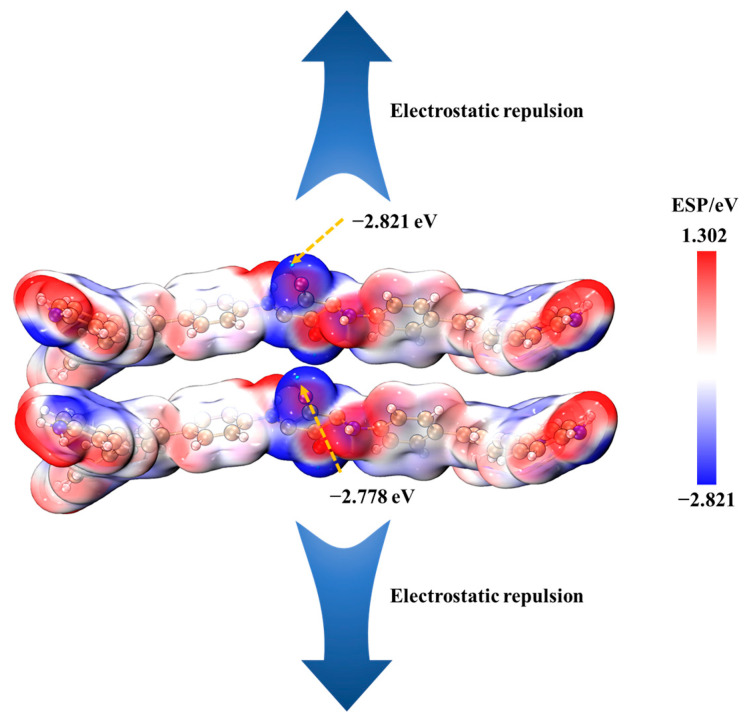
Electrostatic potential of SQA-COF-5.

**Figure 12:**
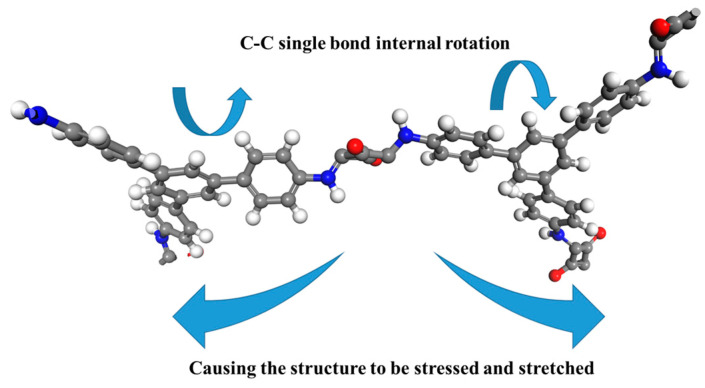
Stress tensile in SQA-COF-5 monolayer.

Through the above analysis of slippage, planarity, and weak interactions, it is evident that SQA-COF-1 and MLA-COF have stronger stacking effects than SQA-COF-5. Furthermore, we calculated the electrostatic potential (ESP) of SQA-COF-5, as shown in Figure 11. The negative potential is concentrated on the oxygen atoms in the single layer, and the positive potential is concentrated nearby. The large electrostatic repulsion between layers can weaken the π-π interaction, making slippage more likely to occur. On the other hand, the internal rotation of the C-C single bond in the SQA-COF-5 nodal unit, as shown in Figure 12, is under tensile stress in the single-layer structure, and the arrangement between layers is irregular, leading to poor stacking.

### 2.3. Macroscopic Performance about Photocatalysis

**Figure 13:**
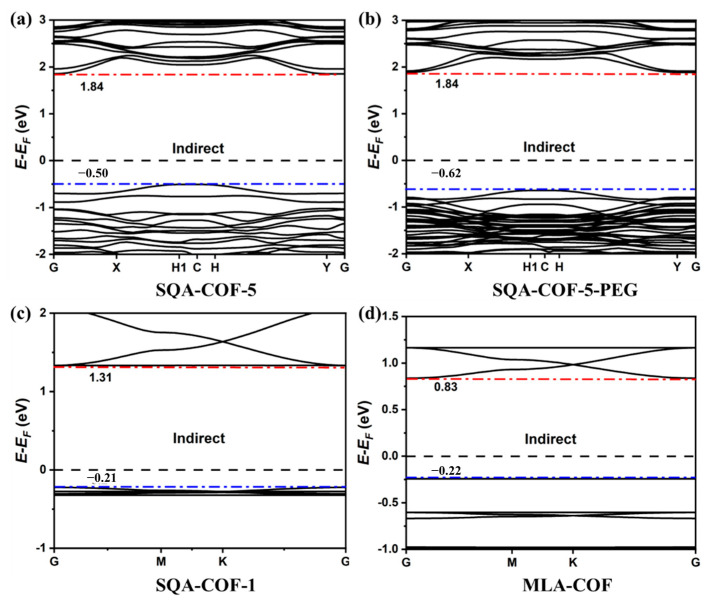
Band structures of (**a**) SQA-COF-5, (**b**) SQA-COF-5-PEG, (**c**) SQA-COF-1, and (**d**) MLA-COF.

Furthermore, we investigated the relationship between the weak interactions and macroscopic performance about photocatalysis. It is important to guide the design of high-performance photocatalysis materials, as it can bridge the gap between microcosmic weak interactions and macroscopic properties. The polarization electric field strength of the materials is as shown in Table 4. It can be seen that the introduction of PEG and the introduction of units with better planarity both enhance the macroscopic performance of the material. Table 5 shows the macroscopic property parameters calculated for SQA-COF-5, SQA-COF-5-PEG, SQA-COF-1, and MLA-COF. Firstly, in terms of bandgap (*E*_g_), the band diagram (Figure 13) shows that the four Z-COFs are indirect bandgap semiconductors, with SQA-COF-1 and MLA-COF displaying smaller bandgaps than SQA-COF-5 and SQA-COF-5-PEG, indicating stronger light excitation capabilities, which are beneficial for the transport of charge carriers. Secondly, the dielectric constants (*ε*_r_) of SQA-COF-1 and MLA-COF are smaller than those of SQA-COF-5 and SQA-COF-5-PEG, indicating that enhancing π-π interactions is beneficial for improving the polarization capability of COFs. Next, the effective masses of holes and electrons, $m_{h}^{*}$ and $m_{e}^{*}$, indicate the transport capabilities of hole and electron carriers, respectively. The smaller the values of $m_{h}^{*}$ and $m_{e}^{*}$, the stronger the transport capability. SQA-COF-1 has the smallest effective mass for holes, indicating the strongest hole carrier transport capability, while MLA-COF has the smallest effective mass for electrons, indicating the strongest electron carrier transport capability. It can be seen that enhancing π-π interactions can improve the transport capability of charge carriers. Finally, from the perspective of exciton binding energy (*E*_b_), in enhancing π-π interactions, the exciton binding energies of SQA-COF-1 and MLA-COF are very small, indicating that their excitons are more likely to separate and less likely to recombine.

## 3. Calculation Methods

The crystal cell structures of Z-COFs were established using Materials Studio 7.0 software, and the structures were optimized using the DFTB+ module under the COMPASS II force field. Before calculating slippage, the optimized primitive cell structures were expanded to create a 2 × 2 × 2 supercell. Subsequently, based on the supercell, a layer of the supercell was translated along the *b*-axis by a distance of 0.25 times the *b*-axis length, and the parameters after slippage were then calculated.

Planarity and the independent gradient model based on Hirshfeld partition (IGMH) were assessed using the Multiwfn 3.8 (dev.) package [22]. Moreover, the Vienna Ab initio Simulation Package (VASP) 5.4.4 [23,24,25,26] was utilized to calculate macroscopic performance via the common plane wave method combined with the PBE functional in the VASP software, with a k-point density of 1 × 1 × 6. More calculation details are shown in the supporting information.

## 4. Conclusions

In this work, we selected four different structures of Z-COFs, including SQA-COF-5, SQA-COF-5-PEG, SQA-COF-1, and MLA-COF, and mainly studied the interlayer interactions of Z-COFs and the impact of interlayer interactions on the macroscopic performance of photocatalysis. From the established primitive cell crystal model parameters, it can be seen that SQA-COF-1 and MLA-COF have a higher symmetry P6/m space group, smaller interlayer spacing, and can better exert the anchoring effect of π-π stacking. Further research on the (001) crystal plane slippage of these four types of Z-COFs shows that the change in the *c*-axis interlayer spacing is the most significant. However, SQA-COF-1 and MLA-COF, which have better π-π stacking interactions, exhibit much smaller changes in interlayer spacing (*c*-axis) than SQA-COF-5. The physical barrier effect formed by PEG inside the channels of SQA-COF-5-PEG and the hydrogen bond coordination effect between PEG and the channel structure can effectively suppress slippage, providing an effective strategy for inhibiting interlayer slippage of SQA-COF-5. In addition, calculations of planarity and weak interactions also demonstrate that SQA-COF-1 and MLA-COF have very strong π-π interactions between layers, which can stabilize the COF framework. We explained the weak interlayer action of SQA-COF-5 through electrostatic potential calculations and the internal rotation of C-C single bonds, which is due to strong electrostatic repulsion and structural stress stretching caused by the internal rotation, resulting in poor stacking of SQA-COF-5. Finally, the calculation of macroscopic properties for photocatalysis indicates that enhancing π-π interactions is beneficial for improving the polarization capability and charge carrier transport capability of COFs, and the exciton binding energy is reduced, making them less likely to recombine. We believe that this work can be beneficial to guide the experimental design of new high-performance photocatalytic materials.

## Figures and Tables

**Table 1:** Cell parameters of SQA-COF-5, SQA-COF-5-PEG, SQA-COF-1, and MLA-COF.

Structures	*a* (Å)	*b* (Å)	*c* (Å)	*α* (°)	*β* (°)	*γ* (°)	Space Group
SQA-COF-5	31.742	32.205	3.869	90.760	90.920	122.398	P1
SQA-COF-5-PEG	31.673	32.138	4.064	90.753	90.931	122.477	P1
SQA-COF-1	16.094	16.094	3.428	90.000	90.000	120.000	P6/m
MLA-COF	20.488	20.488	3.427	90.000	90.000	120.000	P6/m

**Table 2:** Supercell parameters of SQA-COF-5, SQA-COF-5-PEG, SQA-COF-1 and MLA-COF before slippage.

Structures	*a* (Å)	*b* (Å)	*c* (Å)	*α* (°)	*β* (°)	*γ* (°)
SQA-COF-5	63.484	64.410	7.737	90.760	90.920	122.398
SQA-COF-5-PEG	63.345	64.277	8.129	90.753	90.931	122.477
SQA-COF-1	32.188	32.188	6.857	90.000	90.000	120.000
MLA-COF	40.976	40.976	6.855	90.000	90.000	120.000

**Table 3:** Supercell parameters of SQA-COF-5, SQA-COF-5-PEG, SQA-COF-1 and MLA-COF after slippage.

	*a* (Å)	*b* (Å)	*c* (Å)	*α* (°)	*β* (°)	*γ* (°)	L1 (Å)	L2 (Å)
SQA-COF-5	63.680	64.175	10.988	90.982	89.597	122.400	16.000	15.812
SQA-COF-5-PEG	/	/	/	/	/	/	/	/
SQA-COF-1	31.601	31.661	7.504	87.948	89.495	120.543	8.000	1.357
MLA-COF	40.846	40.367	7.432	92.438	92.292	119.474	10.000	1.222

Note: L1 is a slip of 0.25 times the *b*-axis length along the *b*-axis, and L2 is the distance of the actual slip after optimization.

**Table 4:** The polarized electric field strength of materials.

Structures	*E* (N/C)
SQA-COF-5	1.43 × 10^22^
SQA-COF-5-PEG	2.13 × 10^22^
SQA-COF-1	5.17 × 10^23^
MLA-COF	9.14 × 10^23^

**Table 5:** Macroscopic property parameters of SQA-COF-5, SQA-COF-5-PEG, SQA-COF-1, and MLA-COF.

Structures	*E*_g_ (eV)	*ε*_r_ (F/m)	$m_{h}^{*}$ (*m*_0_)	$m_{e}^{*}\;(m_{0})$	*E*_b_ (meV)
SQA-COF-5	2.34	4.12	−10.64	7.93	24.89
SQA-COF-5-PEG	2.48	5.21	−15.63	22.02	26.96
SQA-COF-1	1.52	2.98	−20.65	1.26	2.07
MLA-COF	1.05	2.71	−32.81	0.51	0.95

Note: *m*_0_ is the quality of the electron.

## Data Availability

Data are contained within the article and Supplementary Materials.

## Supplementary Materials

The following supporting information can be downloaded at: https://www.mdpi.com/article/10.3390/molecules29122739/s1. Figure S1. Comparison of Cell parameters of SQA-COF-5, SQA-COF-5-PEG, SQA-COF-1, and MLA-COF; Figure S2. Supercell parameters of SQA-COF-5, SQA-COF-5-PEG, SQA-COF-1 and MLA-COF before and after slippage; Figure S3. The basis for the judgment of the IGMH map; Table S1. Specific volume electron energy before and after slippage.
