# Supplementary material for: Interlayer Interactions and Macroscopic Property Calculations of Squaric-Acid-Linked Zwitterionic Covalent Organic Frameworks: Structures, Photocatalytic Carrier Transport, and a DFT Study
Source: Molecules. 2024 Jun 8;29(12):2739. doi: 10.3390/molecules29122739 (PMC11207002; doi:10.3390/molecules29122739)
Supplement: Supplementary file 1 [file molecules-29-02739-s001.zip › molecules-3039349-supplementary.pdf]

Supporting information for

Interlayer Interactions and Macroscopic Property Calculations of Squaric Acid-linked Zwitterionic Covalent Organic frameworks: A DFT study

Gaojie Yan and Xiaojie Zhang *

Hebei Key Laboratory of Functional Polymers, Department of Polymer Materials and Engineering,
Hebei University of Technology, Tianjin 300401, China; 202021501025@stu.hebut.edu.cn
* Correspondence: zhangxj@hebut.edu.cn

1. Calculation details

The concept of specific volume electron energy (E_V) was defined to explore the relationship between interlayer slippage and interactions in Z-COFs.

$$E_V = E/V \tag{S1}$$

In equation S1, E represents the electronic energy in kcal/mol, V represents the unit cell volume in Å^3, and E_V represents the specific volume electron energy in kcal/mol/Å^3. Generally, the smaller the specific volume energy, the less likely the slippage is to occur.

Planarity is an important indicator affecting π-π interactions. Generally, the better the planarity of a molecule, the better its intermolecular π-π stacking. The planarity calculation uses the optimized primitive cell model, and the planarity of the unit cells in SQA-COF-5, SQA-COF-5-PEG, SQA-COF-1 and MLA-COF was examined using Multiwfn 3.8. The program defines two parameters: the molecular planarity parameter (MPP) and the span of deviation from plane (SDP). Equations S2-S5 define MPP and SDP as follows:

$$MPP = \sqrt{\frac{1}{N_{atom}} \sum_i d_i^2} \tag{S2}$$

$$d_i = \frac{|Ax_i + By_i + Cz_i + D|}{\sqrt{A^2 + B^2 + C^2}} \tag{S3}$$

$$d_i^s = \frac{Ax_i + By_i + Cz_i + D}{\sqrt{A^2 + B^2 + C^2}} \tag{S4}$$

$$SDP = d_{\max}^s - d_{\min}^s \tag{S5}$$

In Equation S2, N_{atom} is the number of atoms, d_i represents the distance of an atom from the fitted plane, and $\sum_i d_i^2$ is the sum of the squares of the distances of each atom from the fitted plane. Equation S3 provides the specific formula for calculating the distance d_i of an atom from the fitted plane, where A, B, C, and D are the coefficients of the fitted plane equation. And x_i, y_i and z_i are the coordinates of the atom. Equation S4 defines the signed distance d_i^s, which is used to examine the degree of atomic deviation from the fitted plane, with the absolute value of d_i to give a positive or negative value, where positive indicates above the fitted plane and negative indicates below it. Equation S5 is used to calculate the SDP, where $d_{\max}^s$ is the maximum distance of an atom from the fitted plane, taken as a positive value, and $d_{\min}^s$ is the minimum distance, taken as a negative value.

The macroscopic properties of photocatalytic materials, such as bandgap (E_g), dielectric constant (ε_r), effective mass of holes (m_h^*), effective mass of electrons (m_e^*), and exciton binding energy (E_b), can be used to evaluate macroscopic performance of photocatalysis materials. E_g was calculated using the common plane wave method combined with the PBE functional in the VASP software, with a k-point density of 1×1×6.

Since COFs are hexagonal crystals with anisotropy, the calculation of ε_r (Equations S6-S7), m_h^* (Equation S8), and m_e^* (Equation S9) needs to consider the two perpendicular wave vector

directions of {001} and {110}, represented by "||" and "⊥", respectively. The effective mass is obtained by fitting the band edges (Equation S10), with m_h^* being fitted for the valence band and m_e^* for the conduction band. E_b is then calculated according to Equations S11 and S12.

$$\varepsilon_{r,\|}(\varepsilon_{r,\perp}) = \varepsilon_{\infty} + \varepsilon_{\mathrm{vib}} \tag{S6}$$

$$\varepsilon_r = \sqrt{\varepsilon_{r,\|} \times \varepsilon_{r,\perp}} \tag{S7}$$

$$\frac{1}{m_h^*} = \frac{2}{3m_{h,\perp}^*} + \frac{\varepsilon_{r,\perp}}{3\varepsilon_{r,\|} m_{h,\|}^*} \tag{S8}$$

$$\frac{1}{m_e^*} = \frac{2}{3m_{e,\perp}^*} + \frac{\varepsilon_{r,\perp}}{3\varepsilon_{r,\|} m_{e,\|}^*} \tag{S9}$$

$$E(k) = E(k_0) \pm \frac{h^2}{m_{\|}^*}(k_0 - k_{\|})^2 \pm \frac{h^2}{m_{\perp}^*}(k_0 - k_{\perp})^2 \tag{S10}$$

$$E_b = E_H \frac{\mu}{\varepsilon_r^2} \tag{S11}$$

$$\frac{1}{\mu} = \frac{1}{m_e^*} + \frac{1}{m_h^*} \tag{S12}$$

In equation S6, $\varepsilon_{r,\|}$ is the dielectric constant in the {001} wave vector direction. $\varepsilon_{r,\perp}$ is the dielectric constant in the {110} wave vector direction. ε_{∞} is the contribution of the electron to the dielectric constant, and $\varepsilon_{\mathrm{vib}}$ is the contribution of frequency to the dielectric constant. In Equation S7, ε_r is the total dielectric constant of the material. In Equation S8, m_h^* is the total effective mass of holes. $m_{h,\perp}^*$ is the effective mass of holes in the {110} wave vector direction, and $m_{h,\|}^*$ is the effective mass of holes in the {001} wave vector direction. In Equation S9, m_e^* is the total effective mass of electrons. $m_{e,\perp}^*$ is the effective mass of electrons in the {110} wave vector direction, and $m_{e,\|}^*$ is the effective mass of electrons in the {001} wave vector direction. Equation S10 is the equation for fitting the effective masses of holes and electrons, where "+" represents the conduction band and "-" represents the valence band. $E(k)$ and $E(k_0)$ represent the energies at wave vectors k and k_0, respectively. k_0 is the wave vector at the band extremum. $m_{\|}^*$ is the effective mass in the {001} wave vector direction. $m_{|\perp}^*$ is the effective mass in the {110} wave vector direction. $k_{\|}$ and $k_{\perp}$ are the wave vectors in the {001} and {110} directions, respectively. h is the Planck constant, $6.62607015 \times 10^{-34}$ J·s. Equation S11 is the formula for calculating the exciton binding energy, where E_b is the exciton binding energy; E_{H} represents the energy of the 1s orbital of a hydrogen atom, which is 13.6 eV; μ is the reduced effective mass; ε_r is the dielectric constant. Equation S12 can be used to solve the reduced mass, where μ is the reduced effective mass; m_h^* is the total effective mass of holes, and m_e^* is the total effective mass of electrons.

Furthermore, the shortest transport distance of electrons can be obtained from the exciton binding energy (Equation S13), and the polarization electric field strength E can be solved (Equations S14-S15). In Equation S13, E_b is the exciton binding energy; e is the charge of an electron; ε_0 is the vacuum permittivity, $8.854187817 \times 10^{-12}$ F/m. ε_r is the materials' dielectric constant, and r is the shortest moving distance of the hole and electron. In Equations S14 and S15, E is the electric field strength.

$$E_b = \frac{e^2}{4\pi\varepsilon_0\varepsilon_r r^2} \times r \tag{S13}$$

$$eE = \frac{e^2}{4\pi\varepsilon_0\varepsilon_r r^2} \tag{S14}$$

$$E = \frac{E_b^2\varepsilon_r}{4\pi\varepsilon_0 e^3} \tag{S15}$$

2. Supporting figures and tables

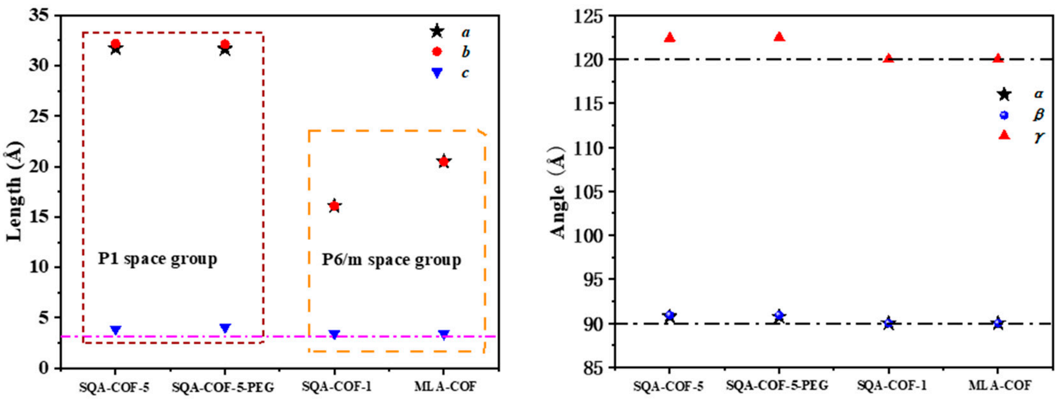

Figure S1. Comparison of Cell parameters of SQA-COF-5, SQA-COF-5-PEG, SQA-COF-1, and MLA-COF.

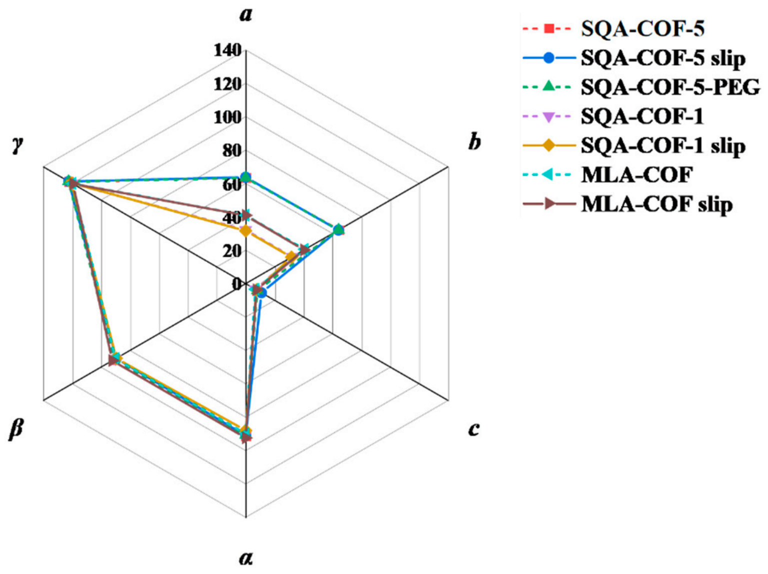

Figure S2. Supercell parameters of SQA-COF-5, SQA-COF-5-PEG, SQA-COF-1 and MLA-COF before and after slippage.

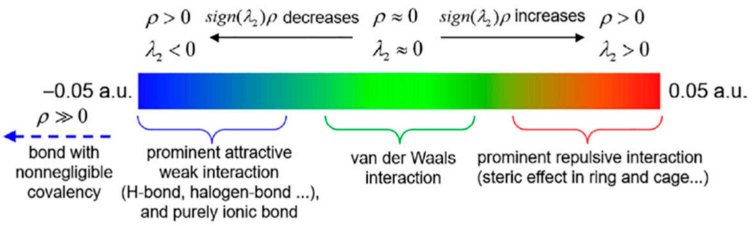

Figure S3. The basis for the judgment of the IGMH map.

Table S1. Specific volume electron energy before and after slippage.

Structures	before slippage			after slippage		
	E (kcal/mol)	*V* (Å^3)	E_v (kcal/mol/Å^3)	*E* (kcal/mol)	*V* (Å^3)	E_v (kcal/mol/Å^3)
SQA-COF-5	15320.46	26700.10	0.574	13116.3	37909.3	0.346
SQA-COF-5-PEG	15241.29	27907.30	0.546	/	/	/
SQA-COF-1	1266.29	6152.43	0.206	2929.361	6458.63	0.454
MLA-COF	2960.964	9967.36	0.297	4642.359	10632	0.437
